# Supplementary material for: The Occurrence of Mycotoxins in Raw Materials and Fish Feeds in Europe and the Potential Effects of Deoxynivalenol (DON) on the Health and Growth of Farmed Fish Species—A Review
Source: Toxins (Basel). 2021 Jun 5;13(6):403. doi: 10.3390/toxins13060403 (PMC8226812; doi:10.3390/toxins13060403)
Supplement: Supplementary file 1 [file toxins-13-00403-s001.zip › Table S1, EU limits.pdf]

Table S1. Legal mycotoxins limits in animal feed ingredients and (fish) feeds set by European Commission.

Undesirable substance	Products intended for animal feed	Maximum content in µg/kg (ppb) relative to a feed with a moisture content of 12 %
DIRECTIVE 2002/32/EC OF THE EUROPEAN PARLIAMENT AND OF THE COUNCIL of 7 May 2002 on undesirable substances in animal feed (EC, 2002)		
Aflatoxin B1		
	Feed materials	20
	Complementary and complete feed	10
COMMISSION RECOMMENDATION of 17 August 2006 on the presence of deoxynivalenol, zearalenone, ochratoxin A, T-2 and HT-2 and fumonisins in products intended for animal feeding (EC, 2006)		
Deoxynivalenol		
	Cereals and cereal products with the exception of maize by-products	8000
	Complementary and complete feedingstuffs with the exception of pigs, calves (< 4 months), lambs and kids	5000
Zearalenone		
	Cereals and cereal products with the exception of maize by-products	2000
Ochratoxin A		
	Cereals and cereal products	250
Fumonisin B1 + B2		
	Maize and maize products	60000
	Complementary and complete feedingstuffs for fish	10000
COMMISSION RECOMMENDATION of 27 March 2013 on the presence of T-2 and HT-2 toxin in cereals and cereal products (EC, 2013)		
T-2 & HT-2 Toxin		
	Cereals and cereal products with the exception of oat bran	500
COMMISSION RECOMMENDATION of 15 March 2012 on the monitoring of the presence of ergot alkaloids in feed and food (EC, 2012)		
Ergot alkaloids		
	Feed containing unground cereals	1000
