# Supplementary material for: The Occurrence of Mycotoxins in Raw Materials and Fish Feeds in Europe and the Potential Effects of Deoxynivalenol (DON) on the Health and Growth of Farmed Fish Species—A Review
Source: Toxins (Basel). 2021 Jun 5;13(6):403. doi: 10.3390/toxins13060403 (PMC8226812; doi:10.3390/toxins13060403)
Supplement: Supplementary file 1 [file toxins-13-00403-s001.zip › Table S2, Inclusion of ingredients in fish diets.pdf]

Table S2. Inclusion of wheat, corn and soybean meal in trout, tilapia, marine fish and carp diets.

Country, year	Wheat	Corn[1]	Soybean meal
Trout			
UK, 2008	10		15
France, 2009	5–10 (7.5)	5–8 (6.5)	10–15 (12.5)
Greece, 2009	5–15 (10)	5–12 (8.5)	10–35 (22.5)
Norway, 2010	12		12
**Average inclusion[2]**	**10.6 %**	**7.5%**	**15.5%**
Tilapia			
Mexico 2009	15–20 (17.5)		30
Venezuela 2008	34		23
Ecuador 2009		20–30 (25)	40
Egypt 2008		30–40 (35)	20-25 (22.5)
Peru 2008		30	40
China 2008			0–25 (12.5)
Taiwan 2007			30–35 (32.5)
Vietnam 2008			30-60 (45)
USA 2008			30-35 (32.5)
**Average inclusion**	**19.9%**	**30 %**	**30.9 %**
Marine fish			
France 2009	5 – 10 (7.5)	10 – 18 (14)	15- 25 (20)
Greece 2009	5 – 15 (10)	5 – 12 (8.5)	10- 35 (22.5)
Spain 2009	1 – 5 (2.5)	4	
UK 2008	10		15
**Average inclusion**	**7.6 %**	**8.8 %**	**19.2 %**
**Carp[3]**			
China 2008	0-25 (13)	0-25 (13)	0-25 (13)
Egypt 2008	10-25 (17.5)	10-25 (17.5)	20-25 (22.5)
India 2006/7	50	50	5
**Average inclusion**	**27%**	**27%**	**13.5 %**

[1]Corn data refers to corn gluten meal in case of trout and marine species.

[2]Average inclusion was estimated taking into account the average/country.

[3]Wheat data refers to wheat bran in case of carp.
