# Supplementary material for: The Occurrence of Mycotoxins in Raw Materials and Fish Feeds in Europe and the Potential Effects of Deoxynivalenol (DON) on the Health and Growth of Farmed Fish Species—A Review
Source: Toxins (Basel). 2021 Jun 5;13(6):403. doi: 10.3390/toxins13060403 (PMC8226812; doi:10.3390/toxins13060403)
Supplement: Supplementary file 1 [file toxins-13-00403-s001.zip › Table S3. Limit of Detection and Limit of Quantification.pdf]

Table S3. Limit of Detection (LOD) and Limit of Quantification (LOQ) for all mycotoxins detected in wheat, corn, soybean meal and aquafeeds.

**Mycotoxin**	**LOD (µg/kg)**	**LOQ (µg/kg)**
Aflatoxin B_1 (AFB_1)	0.13	0.43
Aflatoxin B_2 (AFB_2)	0.54	1.80
Aflatoxin G_1 (AFG_1)	0.15	0.48
Aflatoxin G_2 (AFG_2)	0.15	0.49
Ochratoxin A (OTA)	2.08	6.90
Ochratoxin B	2.22	7.32
Citrinin	0.31	1.02
Deoxynivalenol (DON)	4.35	14.35
3-acetyl-deoxynivalenol (3-AcDON)	2.30	7.55
15-acetyl-deoxynivalenol (15-AcDON)	1.80	5.92
DON-3-Glucoside (DON3Glc)	10.98	36.21
Nivalenol (NIV)	49.92	164.73
Fusarenon X (FX)	2.51	8.30
Beauvericin (BEA)	0.47	1.55
Moniliformin (MON)	1.59	5.23
Fusaric acid (FA)	2.38	7.84
T-2 toxin	0.75	2.48
HT-2 toxin	3.84	12.67
Diacetoxyscirpenol (DAS)	1.52	5.02
Neosolaniol (NEO)	1.82	6.02

Fumonisin B_1 (FB_1)	20.63	68.09
Fumonisin B_2 (FB_2)	1.82	6.01
Fumonisin B_3 (FB_3)	5.00	16.49
Zearalenone (ZEN)	2.57	8.48
Zearalanone	3.46	11.42
Patulin	16.84	55.56
Mycophenolic acid	1.02	3.37
Roquefortine C	1.78	5.86
Penicillic acid	7.43	24.51
Citreoviridin	2.57	8.48
Wortmannin	0.77	2.55
Gliotoxin	5.66	18.69
Sterigmatocystin	0.19	0.61
Cyclopiazonic acid	0.99	3.27
Verruculogen	0.34	1.10
Ergometrin(in)e	0.58	1.91
Ergotamin(in)e	0.51	1.67
Ergocristin(in)e	2.94	9.71
Ergosin(in)e	1.16	3.84
Ergocryptin(in)e	0.81	2.67
Lysergol	0.46	1.52
Methylergonovine	0.05	0.16
Alternariol	1.39	4.60
